# Evaluation of Selected Serum Adipocytokines in Patients with Relapsing–Remitting Multiple Sclerosis Treated with Immunomodulatory Second-Line Drugs

**DOI:** 10.3390/ijms26168070

**Published:** 2025-08-21

**Authors:** Bożena Adamczyk, Natalia Morawiec, Robert Kwinta, Michał Rakoca, Sławomir Wawrzyniak, Jolanta Zalejska-Fiolka, Agata Sowa, Ksawier Sawa, Monika Adamczyk-Sowa

**Affiliations:** 1 Department of Neurology, Faculty of Medical Sciences in Zabrze, Medical University of Silesia in Katowice, 41-800 Zabrze, Poland; bozena.m.adamczyk@gmail.com (B.A.); nataliamorawiec007@gmail.com (N.M.); robertkwintaa@gmail.com (R.K.); s91753@365.eum.edu.pl (A.S.);; 2 Department of Neurology, 10th Military Research Hospital and Polyclinic, 85-681 Bydgoszcz, Poland; mrakoca@op.pl (M.R.); swawrzyniak@wp.pl (S.W.); 3 Department of Biochemistry, Faculty of Medical Science in Zabrze, Medical University of Silesia, 40-055 Katowice, Poland; jzalejskafiolka@sum.edu.pl

**Keywords:** multiple sclerosis, adipocytokines, visfatin, adiponectin, resistin, fingolimod, natalizumab

## Abstract

Adipocytokines are involved in the pathogenesis of multiple sclerosis by modulating inflammation, blood–brain barrier function and immune responses, which may affect disease course and treatment outcomes. Our study assessed serum levels of visfatin, adiponectin and resistin in patients with relapsing–remitting multiple sclerosis treated with fingolimod or natalizumab. We examined 49 patients with relapsing–remitting multiple sclerosis and 38 healthy controls. Participants were divided into three groups: patients treated with fingolimod, those treated with natalizumab and the controls. Serum levels of visfatin, adiponectin and resistin were measured. We analyzed correlations with disease duration, treatment duration and body mass index. Adiponectin levels were significantly higher in patients treated with natalizumab compared to those receiving fingolimod and healthy controls (*p* < 0.05). In the fingolimod group, visfatin levels increased with treatment duration. The mean level was 51.27 pg/mL for treatment shorter than eighteen months and 59.12 pg/mL for longer treatment (*p* < 0.05). In the same group, resistin levels correlated positively with body mass index (*p* < 0.05), while visfatin levels showed a negative correlation (*p* < 0.05). Fingolimod may affect adipocytokine levels, which could support patient monitoring. Increased adiponectin in natalizumab-treated patients suggests its possible role in the therapeutic mechanism of the treatment.

## 1. Introduction

Multiple sclerosis (MS) is an autoimmune disease of the central nervous system (CNS) characterized by inflammation, demyelination and neurodegeneration. The exact cause of its development is still unknown. Predisposing factors include environmental agents, genetics, oxidative stress and metabolic disorders [1]. The epidemiology of MS is changing with advances in diagnosis, lifestyle changes and the availability of immunomodulatory therapies. There has been an increase in incidence, with a milder course of the disease in the last 25 years [1,2]. MS affects mainly young adults between the ages of 20 and 40 [2]. Autoreactive lymphocytes play a key role in disease development. In the early stages, inflammation is predominant. It leads to a gradual neurodegeneration [3]. Primary activation of T lymphocytes, induced by Epstein–Barr virus (EBV) infection or contact with myelin antigens, contributes to the development of MS [4]. Activated T-cells migrate to the CNS, where they undergo further activation and differentiation into subpopulations: pro-inflammatory Th1 cells (secreting IFN-γ, TNF-α, IL-2, IL-12, IL-15), anti-inflammatory Th2 cells (IL-4, IL-5, IL-13) and Th17 cells (secreting IL-17, IL-21, IL-22) [5,6,7]. The increased expression of IL-17 in the cerebrospinal fluid (CSF) and blood of MS patients, especially during relapses, suggests a crucial role of this cytokine in disease progression [7]. Chronic inflammation induces activation of oxidative stress enzymes. This leads to damage of proteins, lipids, carbohydrates and nucleic acids, degeneration of mitochondria, dysfunction of ion channels and activation of apoptosis pathways. In the course of the disease, this process initiates neurodegeneration. Chronic hyperactivation of oxidative enzymes can sustain “smoldering” inflammation, contributing to the progression of MS [8].

The correlation between obesity and the degree of disability in MS has been proven. Obesity promotes inflammation and affects the metabolic profile, and lipoprotein and hormone levels, including adiponectin. In obese individuals, adiponectin levels decrease. This has an inhibitory effect on the production of regulatory T-cells and anti-inflammatory cytokines [9,10,11,12]. A genetically determined high body mass index (BMI), associated with a configuration of genes that increase the risk of overweight in the general population, is associated with a higher risk of developing MS. This suggests that obesity and overweight in childhood and early adulthood are important modifiable risk factors for MS [9]. The increase in average body weight in Western countries in recent decades correlated with an increasing incidence of MS, especially among women [13,14]. In addition to storing energy, adipose tissue secretes adipocytokines that regulate energy balance, insulin sensitivity and immune response. They include visfatin, resistin and adiponectin [15,16].

### Adipocytokines in MS

Adipocytokines, bioactive molecules secreted by the adipose tissue, are implicated in the immunopathology of MS. They modulate key neuro-inflammatory and neuroimmune pathways. Visfatin, also known as nicotinamide phosphoribosyltransferase (NAMPT), is primarily secreted by visceral fat [17]. It exists in two forms: intracellular and extracellular. The intracellular form plays a regulatory role in energy biosynthesis. It is involved in oxidation processes in the cell. It takes part in reductive biosynthesis, detoxification and antioxidation. Extracellular forms affect various signaling pathways, including those related to inflammation [18,19,20]. Visfatin promotes inflammatory responses by enhancing cytokine secretion and T-cell activation. Inhibition of NAMPT reduces clinical symptoms in experimental autoimmune encephalomyelitis models [21]. In MS, visfatin enhances the inflammatory response by promoting the secretion of cytokines and modulating T and B lymphocyte function, contributing to the loss of immune tolerance and perpetuation of CNS autoimmunity [17].

Resistin is secreted by the adipose tissue into the circulatory system. It plays an important role in the release of immune factors that stimulate a pro-inflammatory response, such as TNF-α, IL-1b, IL-6, IL-8 and IL-12 [22]. It also has a protective function by shielding cells from oxidative stress [23,24,25]. Resistin acts both as a promoter of inflammation and as a protector against cellular stress. This dual function complicates its potential use as a direct therapeutic target in inflammatory or metabolic diseases. Reducing resistin activity may help alleviate chronic inflammation and insulin resistance. However, lowering its levels could also impair the cellular defense against oxidative damage. Therefore, therapeutic strategies should focus on selective modulation of resistin pathways. Such approaches should aim to inhibit its pro-inflammatory effects while preserving its cytoprotective functions [26]. In MS, elevated resistin levels are associated with increased TNF-α and IL-1β production and decreased Foxp3 expression, indicating a reduction in regulatory T-cell activity. This favors a chronic inflammatory environment and contributes to neurodegeneration, particularly in progressive MS subtypes [17].

Adiponectin is produced by adipose tissue and is found in the highest amount in blood [27]. Its main function is to maintain fatty acid homeostasis and inhibit gluconeogenesis in the liver [28,29]. Adiponectin exerts primarily anti-inflammatory and neuroprotective functions. In MS, adiponectin levels are elevated in the CSF and correlate with disease severity and progression [30]. Adiponectin exerts neuroprotective actions by reducing oxidative stress, decreasing pro-inflammatory cytokines such as IL-6 and TNF-α, and enhancing IL-10 production, thereby promoting immune regulation in glial and neuronal cells exposed to MS-CSF [31].

Patients with MS have elevated levels of adipocytokines, such as resistin and visfatin, and decreased levels of adiponectin [32]. Hietaharju et al. suggest that adipocytokines may be synthesized in the CNS or transported across the blood–brain barrier, contributing to inflammatory processes in MS [33].

The aim of this study was to evaluate the serum levels of selected adipocytokines in patients with relapsing–remitting multiple sclerosis (RRMS) treated with immunomodulatory second-line drugs, compared to healthy controls in relation to gender, age, disease duration, degree of disability, disease activity, presence of lesions on brain MRI and BMI.

## 2. Results

The RRMS group consisted of 49 individuals, while the control group included 38 participants. Women comprised the majority of the study group (65.31%). The average age of patients with RRMS was 36.11 years, and the average age was 39.94 years in the control group. There were no statistically significant differences between the groups in terms of age (*p* = 0.14) or sex distribution (*p* = 0.23) for the RRMS and control groups (Table 1).

The fingolimod (FG) and natalizumab (NT) groups did not significantly differ in any of the assessed demographic or clinical parameters. The average duration of the disease was 7.46 years for the FG group and 5.52 years for the NT group. The longer disease duration in the fingolimod group reflects treatment practices, where fingolimod is often used later in the disease course, while natalizumab is initiated earlier in highly active cases. BMI values were similar in both groups (*p* = 0.61), as were Expanded Disability Status Scale (EDSS) scores (*p* = 0.92) and the annualized relapse rate (ARR) (*p* = 0.21). There were no significant differences in the mean number of gadolinium-enhancing lesions (*p* = 0.49) or T2 lesions on brain MRI (*p* = 0.32) in the FG and NT groups (Table 2).

A significant difference was found in adiponectin levels in the study groups. The serum levels of visfatin and resistin did not differ significantly among the NT, FG and control groups (*p* = 0.961 and *p* = 0.253, respectively) (Table 3).

A post hoc analysis revealed that adiponectin levels were significantly higher in the NT group compared to both the FG and control groups (*p* = 0.000 for both comparisons). No significant difference in adiponectin levels was observed between the FG and control groups (Table 4).

The post hoc analysis did not show any differences between the levels of adiponectin, visfatin and resistin depending on gender (Table 5).

**Figure 1:**
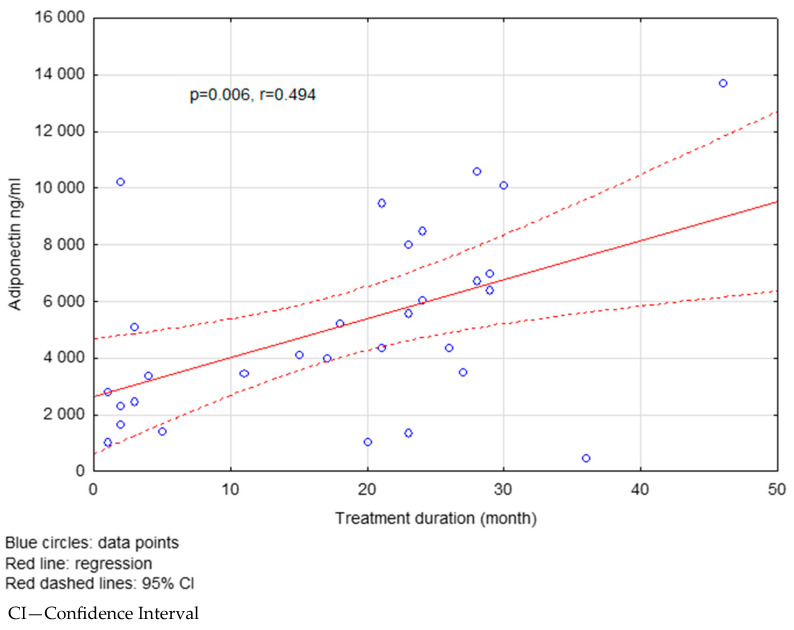
The correlation between the treatment duration and adiponectin levels in the FG group.

Adiponectin levels in the FG-treated group increased with the treatment duration (Figure 1). In the FG-treated group, patients with a treatment duration longer than 18 months had significantly higher serum levels of visfatin (*p* = 0.031) and adiponectin (*p* = 0.022) compared to those treated for less than 18 months. No significant difference was observed in resistin levels (*p* = 0.734) (Table 6).

**Figure 2:**
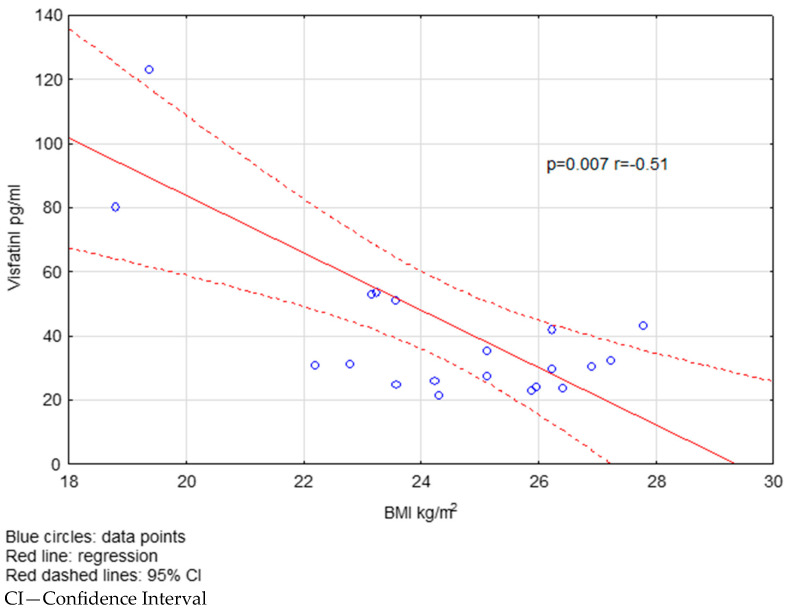
The correlation between visfatin levels and BMI in the FG-treated group.

**Figure 3:**
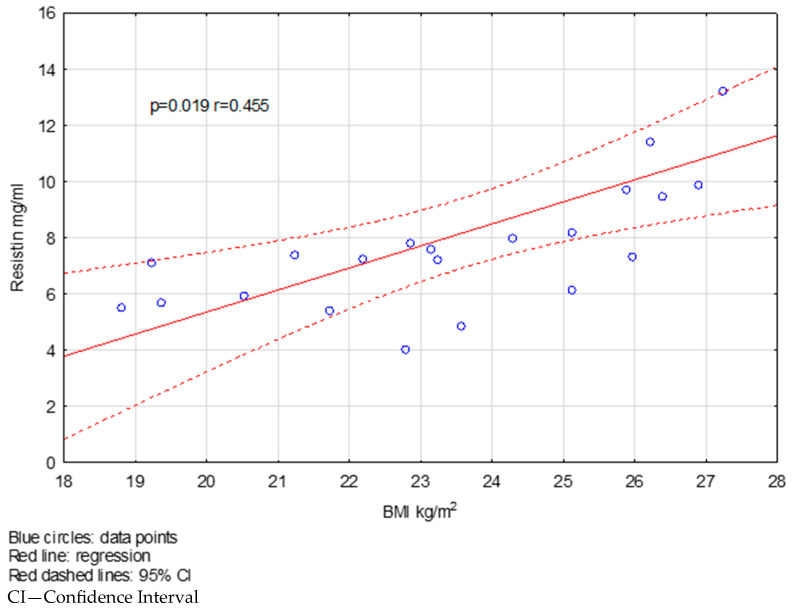
The correlation between resistin levels and BMI in the FG-treated group.

We found a negative correlation between visfatin levels and BMI in the FG group (Figure 2). In the FG-treated group, resistin concentrations correlated positively with BMI (Figure 3). There were no significant correlations in the NT group.

## 3. Discussion

The pathogenesis of MS includes genetic factors, environmental factors and metabolic disorders [34,35]. Recently, a lot of studies have pointed to the important role of adipocytokines in MS, which may be related to the influence of adipose tissue on inflammatory and neurodegenerative processes [16,32,33,36]. Obesity, especially during adolescence, has been identified as a risk factor for MS development. The mechanisms of its influence include changes in adipocytokine profile and chronic inflammation [16,36]. Furthermore, obesity may worsen the course of autoimmune diseases. Adipose tissue cells secrete numerous pro-inflammatory cytokines. The biochemical links between chronic inflammation and the development of autoimmune diseases have been identified. CNS immune cells communicate with systemic immune cells using various transmitters including adipocytokines [37]. Total body fat may be a more important factor in MS inflammation than BMI or the amount of subcutaneous fat [38]. Adipose tissue may be responsible for producing adipocytokines and could affect immune cells [39].

The different effects of fingolimod and natalizumab on adipocytokine levels in our study may be related to their distinct mechanisms of action.

### 3.1. Fingolimod and Adipocytokines

Fingolimod modulates sphingosine-1-phosphate (S1P) receptors on lymphocytes, preventing their egress from lymph nodes and altering immune cell profiles. Recent evidence suggests that fingolimod may upregulate peroxisome proliferator-activated receptor gamma (PPAR-γ), a nuclear receptor involved in anti-inflammatory responses and adipokine regulation [40]. PPAR-γ activation is known to increase adiponectin expression and reduce inflammatory cytokine production, which may partially explain the correlations observed in our fingolimod-treated group [41]. Visfatin is secreted by activated immune cells and exerts strong pro-inflammatory effects by inducing cytokines such as IL-6 and TNF-α. It is also involved in NAD^+^ biosynthesis, linking it to cellular stress responses [17]. Fingolimod has been shown to affect oxidative stress pathways [42]. It may indirectly modulate visfatin expression by reducing immune activation.

Considering the data on the role of visfatin in the pathomechanism of MS, it is possible to conclude that patients with MS have elevated serum levels of visfatin compared to healthy controls. These findings tended to correlate with increased levels of other pro-inflammatory adipocytokines, such as resistin, and decreased adiponectin concentrations [17]. We observed an increase in visfatin levels with the duration of the therapy and its negative correlation with BMI in the group of patients treated with FG. Visfatin is an adipocytokine with pro-inflammatory and metabolic effects. Its elevated levels have been observed in autoimmune diseases, including MS [17,20]. The increase in visfatin in patients treated with FG may be observed due to the drug’s effects on lipid and carbohydrate metabolism, although the exact mechanism remains unclear. There are reports suggesting that visfatin stimulates the production of pro-inflammatory cytokines (TNF-α, IL-6, IL-1β) and may contribute to an increased inflammation process in MS [10,17,43]. Our findings suggest that the effect of visfatin may be therapy-dependent, which requires further longitudinal studies.

Another important observation is the positive correlation between resistin levels and BMI in the FG-treated group. Resistin, like visfatin, has pro-inflammatory effects and is associated with insulin resistance, obesity, diabetes, atherosclerosis and chronic inflammation [17,44,45,46]. Insulin resistance is more common in MS patients and may be related to persistent inflammation or low physical activity [47]. Furthermore, it was observed that patients with insulin resistance scored higher on the EDSS scale than those without insulin resistance [48]. Previous studies have demonstrated elevated resistin levels in patients with active MS, suggesting its involvement in mechanisms associated with chronic low-grade inflammation [17,49,50]. Our results suggest a potential relationship between BMI and resistin levels among FG-treated patients, which may be relevant for monitoring metabolic aspects of therapy.

### 3.2. Natalizumab and Adipocytokines

Natalizumab limits leukocyte trafficking across the blood–brain barrier by blocking α4-integrin, which may differentially impact systemic adipokine expression [51]. We found increased levels of adiponectin in the NT group, which may indicate a beneficial effect of this drug on the regulation of inflammatory processes [52]. Adiponectin exhibits anti-inflammatory and protective effects against insulin resistance. Its increase in MS may be linked to mechanisms that limit disease progression [32,53]. Previous studies have shown that peroxisome proliferator-activated receptor gamma (PPAR-γ) activation regulates adiponectin secretion and may influence the course of MS by reducing the pro-inflammatory response of glial cells [52,54]. There is a lack of data on the mechanism by which NT may increase adiponectin levels. It is known that the two substances may act synergistically through their antioxidant and anti-inflammatory properties [55,56].

### 3.3. Clinical and Radiological Parameters and Adipocytokines

We have not observed any significant correlations between adipocytokines’ levels and clinical or radiological indicators of MS progression (ARR, EDSS and MRI changes). The reason for this may be that BMI and total body fat play a more complex role in adipocytokine regulation than clinical parameters alone. It is important to note that BMI is not always a good indicator of metabolic obesity and that total body fat may be more relevant to assessing the effects of adipocytokines on MS [38]. In the context of future studies, it will be important to consider additional metabolic parameters, such as insulin, glucose levels and lipid profile, which could provide further detailed information on the effects of adipocytokines on MS [57,58].

Adiponectin, visfatin and resistin show potential as biomarkers in MS. Adiponectin has anti-inflammatory and neuroprotective effects, enhancing regulatory T-cell function and reducing oxidative stress in central nervous system cells, which may inform prognosis or therapeutic responsiveness [59,60]. Elevated serum levels of visfatin and resistin correlate with increased pro-inflammatory cytokines and greater clinical disability, suggesting that their trajectories could signal active disease or suboptimal treatment response [60,61]. Longitudinal monitoring of these adipocytokines could support treatment selection and help in predicting disease progression or therapeutic efficacy in patients with RRMS.

Although our findings suggest associations between treatment duration and adipocytokine levels, it is important to note that the study was cross-sectional. Therefore, we cannot infer causality or confirm longitudinal changes in biomarker levels. In addition, the relatively small number of patients treated with natalizumab may limit the statistical power to detect more subtle group differences. These limitations highlight the need for future longitudinal studies with larger, well-balanced cohorts to evaluate the dynamic changes in adipocytokine profiles and confirm their relevance for monitoring treatment response and disease progression in multiple sclerosis.

## 4. Materials and Methods

This prospective study enrolled 87 subjects, including 49 patients diagnosed with RRMS treated with second-line therapies and 38 healthy controls. The inclusion criteria were as follows: patients with RRMS diagnosed according to the 2017 McDonald criteria treated with FG or NT, ability to collect 10 mL of venous blood, age ≥ 18 years, written consent to participate in the study and no relapses in the preceding 6 months. The individuals in the control group were healthy volunteers, non-smokers with normal BMI values (18.5–24.9 kg/m^2^). The controls were included based on self-reported medical history, absence of chronic diseases, no current medication use, confirmation of normal BMI and non-smoking status during screening interviews. The exclusion criteria included the following: lack of consent to participate in the study, coexistence of other autoimmune diseases, use of other drugs and dietary supplements, and being smokers. Study participants were divided into three groups: FG-30 patients treated with FG (0.5 mg 1x/d p.o.), NT-19 patients treated with NT (300 mg every 4 weeks i.v.) and the control group—38 healthy controls. The blood samples were collected from the participants. After centrifugation, the serum was frozen (−80 °C) until the assays. The concentrations of visfatin, adiponectin and resistin were tested using the ELISA method (Human Quantikine^®^ ELISA Kits, R&D Systems Inc., Minneapolis, MN, USA). Demographic and clinical data, including the onset and duration of the disease, clinical presentation of MS, type of treatment, relapsing activity expressed by ARR, degree of disability according to the EDSS scale and radiological activity, were obtained from the National Health Service database, neurology outpatient clinic and neurology department records. The parameters of descriptive statistics are presented as an arithmetical mean and standard deviation, and qualitative variables as percentages. Homogeneity of continuous variables was analyzed using the ANOVA test (for a normal distribution) or the Kruskal–Wallis test (for a non-normal distribution). For significant differences, post hoc analysis (Tukey’s test with Bonferroni correction) was used. For comparisons between two groups, Student’s *t*-test or the Mann–Whitney U test was used. Correlations were assessed with Pearson’s R coefficient. A significance level of *p* < 0.05 was assumed. Calculations were performed in Statistica 12 PL (StatSoft Inc., Tulsa, OK, USA).

The study was conducted in accordance with the ethical standards of the institutional research committee and with the 2013 revision of the Declaration of Helsinki. The protocol was approved by the Bioethics Committee of the Medical University of Silesia in Katowice (decision no. BNW/NWN/0052/KB1/61/I/23/24).

## 5. Conclusions

Our study revealed that natalizumab treatment is associated with significantly elevated serum adiponectin levels in patients with RRMS, which may reflect its anti-inflammatory and neuroprotective properties. In contrast, fingolimod treatment appears to influence adiponectin, visfatin and resistin concentrations. Our findings suggest that immunomodulatory therapies may differentially affect adipocytokine profiles and that BMI may modulate these effects, particularly in patients receiving fingolimod. The clinical relevance of these observations lies in the potential role of adipocytokines as biomarkers of treatment response or disease progression. However, limitations such as the relatively small sample size and cross-sectional design should be acknowledged. Future studies with larger cohorts and longitudinal follow-up are needed to confirm these associations and explore the mechanistic links between adipocytokines, treatment effects and MS pathophysiology.

## Figures and Tables

**Table 1:** General characteristics of the study groups.

	RRMS	Control	*p*
N	49	38	0.32
Age (years)	36.11 ± 11.18	39.94 ± 12.03	0.14
Sex (% women)	65.31	76.31	0.23

RRMS—relapsing–remitting multiple sclerosis. The data are presented as mean ± standard deviation or percentage. Comparisons were made using Student’s *t*-test (for normally distributed variables), Mann–Whitney U test (for non-normal distributions) and chi-squared test (for categorical variables).

**Table 2:** Demographic and clinical characteristics of the study groups.

	FG	NT	*p*
Disease duration (years)	7.46 ± 4.34	5.52 ± 3.23	0.10
BMI (kg/m^2^)	23.47 ± 2.41	23.93 ± 3.82	0.61
EDSS (points)	3.28 ± 1.03	3.11 ± 1.03	0.92
ARR (N)	0.34 ± 0.61	0.32 ± 0.53	0.21
Mean number of Gd(+) lesions on brain MRI (N)	0.11 ± 0.42	0.17 ± 0.71	0.49
Mean number of T2 lesions on brain MRI (N)	19.30 ± 1.65	18.52 ± 2.27	0.32

FG—RRMS patients treated with fingolimod; NT—RRMS patients treated with natalizumab; EDSS—Expanded Disability Status Scale; ARR—annualized relapse rate. The data are presented as mean ± standard deviation. Group comparisons were performed using Student’s *t*-test or Mann–Whitney U test depending on data distribution.

**Table 3:** Comparison of serum levels of selected adipocytokines in NT, FG and control groups.

	NT	FG	Control	*p*
N	19	30	38	
Visfatin (pg/mL)	54.58 ± 40.26	56.75 ± 41.72	48.80 ± 31.52	0.961
Adiponectin (ng/mL)	9372.02 ± 4003.78	5141.65 ± 3362.47	5145.54 ± 2959.85	0.001
Resistin (ng/mL)	10.34 ± 6.64	7.68 ± 3.69	7.43 ± 3.08	0.253

FG—RRMS patients treated with fingolimod; NT—RRMS patients treated with natalizumab. Data are presented as mean ± standard deviation. The differences among groups were analyzed using one-way ANOVA or the Kruskal–Wallis test, depending on distribution. For significant results, post hoc analysis was performed.

**Table 4:** Post hoc analysis for adiponectin in the FG, NT and control groups.

Group	FG	NT	Control
FG	x	*p* = 0.000	NS
NT	*p* = 0.000	x	*p* = 0.000
Control	NS	*p* = 0.000	x

FG—RRMS patients treated with fingolimod; NT—RRMS patients treated with natalizumab; NS—no statistical significance; statistical significance at *p* ≤ 0.05. Post hoc analysis was conducted using Tukey’s test (after ANOVA) or Dunn’s test with Bonferroni correction (after Kruskal–Wallis test).

**Table 5:** Comparison of serum levels of selected adipocytokines in RRMS groups and controls depending on gender.

	Women with RRMS	Men with RRMS	Women —Control Group	Men —Control Group	*p*
N	32	17	29	9	
Visfatin (pg/mL)	54.11 ± 40.01	59.29 ± 43.16	48.68 ± 33.34	49.19 ± 26.52	0.733
Adiponectin (ng/mL)	6587.80 ± 3866.66	6983.77 ± 4686.89	5303.22 ± 2980.45	4637.46 ± 3007.82	0.397
Resistin (ng/mL)	8.46 ± 5.37	9.18 ± 4.82	7.52 ± 3.27	7.14 ± 2.57	0.447

RRMS—RRMS patients treated with fingolimod and natalizumab. The data are presented as mean ± standard deviation. Comparisons between sex-specific subgroups were performed using ANOVA or the Kruskal–Wallis test based on data distribution.

**Table 6:** Comparison of serum levels of selected adipocytokines in the FG-treated group depending on the duration of treatment.

Treatment Duration (Months)	<18	>18	*p*
Visfatin (pg/mL)	51.27 ± 43.49	59.12 ± 39.22	0.031
Adiponectin (ng/mL)	5916.72 ± 4306.89	7307.55 ± 3975.48	0.022
Resistin (ng/mL)	9.37 ± 6.50	8.25 ± 4.03	0.734

The data are presented as mean ± standard deviation. Comparisons were performed using Student’s *t*-test or Mann–Whitney U test, depending on data distribution.

## Data Availability

Data is contained within the article.
